# Supplementary material for: The interplay between movement, morphology and dispersal in Tetrahymena ciliates
Source: PeerJ. 2019 Dec 17;7:e8197. doi: 10.7717/peerj.8197 (PMC6924321; doi:10.7717/peerj.8197)
Supplement: Supplemental Information 1 — Details about genotypes used, the experimental system and the trajectory reconstruction. [file peerj-07-8197-s001.docx]

Supplementary information for:

**The interplay between movement, dispersal and morphology in *Tetrahymena* ciliates**

Frank Pennekamp^1,2^, Jean Clobert^3^ & Nicolas Schtickzelle^1^

^1^Earth and Life Institute & Biodiversity Research Centre, Université catholique de Louvain, Croix du Sud 4, L7.07.04, 1348 Louvain-la-Neuve, Belgium

^2^Present address: Institute of Evolutionary Biology and Environmental Studies, University of

Zurich, Winterthurerstrasse 190, 8057 Zurich, Switzerland

^3^  Station d’Ecologie Théorique et Expérimentale, CNRS, 09200 Moulis, France

## *Tetrahymena thermophila* genotypes used and culture conditions

In this study, we used a set of 44 genetically distinct genotypes (synonyms: clonal lines, strains) of the ciliate protist *Tetrahymena thermophila* that have a different history in terms of geographical location at date of isolation (Pennekamp et al. 2014, see Table S1). We previously showed they differ in several life history traits such as growth rate, maximum cell density and survival under starvation conditions (Fjerdingstad et al. 2007, Pennekamp 2014). Cells only reproduced clonally because all cells from one genotype share the same mating type, preventing sexual reproduction through conjugation (Collins 2012); this ensures stability of the genotype over the duration of our study.

After defrosting cells from stock cultures kept in suspended animation in liquid nitrogen (Altermatt et al. 2015), the 44 genotypes were maintained under standard culture conditions before and during the experiment: axenic liquid culture in a nutrient medium (broth consisting of 2% Proteose peptone and 0.2% yeast extract [Becton Dickinson], diluted in ultrapure water), kept at constant 27°C temperature in a light controlled incubator with a 14:10 hours light/dark cycle. Culture stocks were renewed every 10 days by inoculating a 2 mL sample of fresh medium with 100 µL of culture and maintained in 2 mL multi-well plates (CELLSTAR® multiwell plates, Ref. 662102 from Greiner BioOne, Belgium). All manipulations of axenic cultures were conducted under sterile conditions in a laminar flow hood (Ultrasafe 218 S, Faster, Italy).

## Experimental design with two patch dispersal systems

We quantified dispersal and movement behaviour of *T. thermophila* cells using the same standardized two patch system developed in our previous work (Fjerdingstad et al. 2007, Schtickzelle et al. 2009, Chaine et al. 2010, Pennekamp et al. 2014) (Figure S1).

In addition to the strict control of all our *T. thermophila* culture conditions, two standardization steps were performed prior to the experiment. First, a pre-culture of each genotype was started from the stock by transferring 100 µL of culture into 2 mL of fresh nutritive medium (2% Proteose peptone and 0.2% yeast extract [Becton Dickinson], diluted in ultrapure water) on a 24 well plate (CELLSTAR® multiwall plate, Ref. 662102 from Greiner BioOne, Belgium) and allowed to grow exponentially for 4 days to synchronize populations to the logarithmic phase of population growth (Collins 2012). Second, at the end of this synchronization phase, cell density was estimated for each genotype, and new cultures, to be used for the experiment, were launched at an equal starting density of 10000 cells/mL in culture flasks (CELLSTAR® Cell Culture Flask 50 mL, Ref. 690175 from Greiner BioOne, Belgium). These cultures grew for three days allowing them to reach sufficiently high cell densities for the experiment.

Each dispersal system consisted of two standard 1.5 mL microtubes connected by a silicon pipe (length: 17 mm; external diameter: 6 mm; Ref. 228-0709 from VWR, Belgium). The dispersal system was filled prior to the experiments with 3 mL of the standard nutritive medium through one tube to ensure fluid transition between the tubes and a connecting pipe free of air bubbles. The system was then closed by placing a clamp in the middle of the connecting pipe. To start the experiment, cells were inoculated into the “start” tube of the system at a density of density of 300000 cells / mL (i.e. 450000 cells for a 1.5 mL volume) and the tube content was homogenized to encourage the cells to move freely throughout the start tube. After 30 minutes of acclimation to the new medium, the clamp closing the connecting pipe was removed and cells could freely disperse between the two tubes for 6 h. After these 6 h, the pipe was clamped again, and five independent samples taken from the “start” and “target” tubes after culture homogenization. Each sample was loaded into the chamber of a counting slide (Precision cell 301890, Vacutest Kima, Italy).

## Reconstructing movement trajectories from videos

We developed for this study a workflow to extract movement trajectories from digital videos in a standardized and automated fashion, which was later transformed into the R package BEMOVI (Pennekamp et al. 2015). We customized the ParticleTracker plug-in for ImageJ software, originally aimed at tracking intracellular movements of cell structure (<http://mosaic.mpi-cbg.de/ParticleTracker/>). The ParticleTracker links each position of a given cell, as recorded on every frame of the video, into a unique trajectory of time-stamped X and Y coordinates, which are output as text files. The 25 frames per second acquisition speed for our videos guarantees correct assembly of positions into individual trajectories even when many cells are tracked simultaneously. ParticleTracker’s feature point tracking algorithm is described in detail in Sbalzarini & Koumoutsakos (2005) and has several powerful features: 1) tracking of many individuals simultaneously is feasible and due to low computational requirements longer video sequences may be analysed; 2) the plug-in deals with unrestricted viewing fields, i.e. cells may leave and enter the video because the viewing field is not physically bounded; 3) in case the algorithm is unable to retain the identity of a given cell because of collisions between tracked cells or with artefacts on the video such as dust, it acts conservatively by terminating the current trajectories and assigning new trajectory identities when cells are again separated.

Home-made SAS scripts (www.sas.com) were used to read the raw trajectory data extracted by ParticleTracker (Figure S2). 49% of the recorded trajectories (258,592 out of 525,328) were discarded because they lasted less than 1 s or had a total net displacement (i.e. the bee line distance between the start and the end position of the trajectory) less than 50 µm (corresponding to one body length of an average *Tetrahymena* cell); such trajectories correspond to non-moving cells. For analysis with continuous time movement models (Fleming et al. 2014, Gurarie et al. 2017), which is highly computationally demanding, we then subsampled 23 trajectories for each genotype x replicate x tube combination, resulting in 6072 trajectories sampled from a total of 266,736 trajectories of moving cells.

## References

Altermatt, F. et al. 2015. Big answers from small worlds: a user’s guide for protist microcosms as a model system in ecology and evolution. - Methods Ecol. Evol. 6: 218–231.

Chaine, A. S. et al. 2010. Kin-based recognition and social aggregation in a ciliate. - Evolution 64: 1290–1300.

2012. Tetrahymena thermophila (K Collins, Ed.). - Academic Press.

Fjerdingstad, E. J. et al. 2007. Evolution of dispersal and life history strategies – Tetrahymena ciliates. - BMC Evol. Biol. 7: 133.

Fleming, C. H. et al. 2014. From Fine-Scale Foraging to Home Ranges: A Semivariance Approach to Identifying Movement Modes across Spatiotemporal Scales. - Am. Nat. 183: E154–E167.

Gurarie, E. et al. 2017. Correlated velocity models as a fundamental unit of animal movement: synthesis and applications. - Mov. Ecol. 5: 13.

Pennekamp, F. 2014. Swimming with ciliates: dispersal and movement ecology of Tetrahymena thermophila, PhD thesis.

Pennekamp, F. et al. 2014. Dispersal propensity in *Tetrahymena thermophila* ciliates—a reaction norm perspective. - Evolution 68: 2319–2330.

Pennekamp, F. et al. 2015. BEMOVI, software for extracting behavior and morphology from videos, illustrated with analyses of microbes. - Ecol. Evol. 5: 2584–2595.

Sbalzarini, I. F. and Koumoutsakos, P. 2005. Feature point tracking and trajectory analysis for video imaging in cell biology. - J. Struct. Biol. 151: 182–195.

Schtickzelle, N. et al. 2009. Cooperative social clusters are not destroyed by dispersal in a ciliate. - BMC Evol. Biol. 9: 251.
