# Supplementary figures and images for: The interplay between movement, morphology and dispersal in Tetrahymena ciliates
Source: PeerJ. 2019 Dec 17;7:e8197. doi: 10.7717/peerj.8197 (PMC6924321; doi:10.7717/peerj.8197)

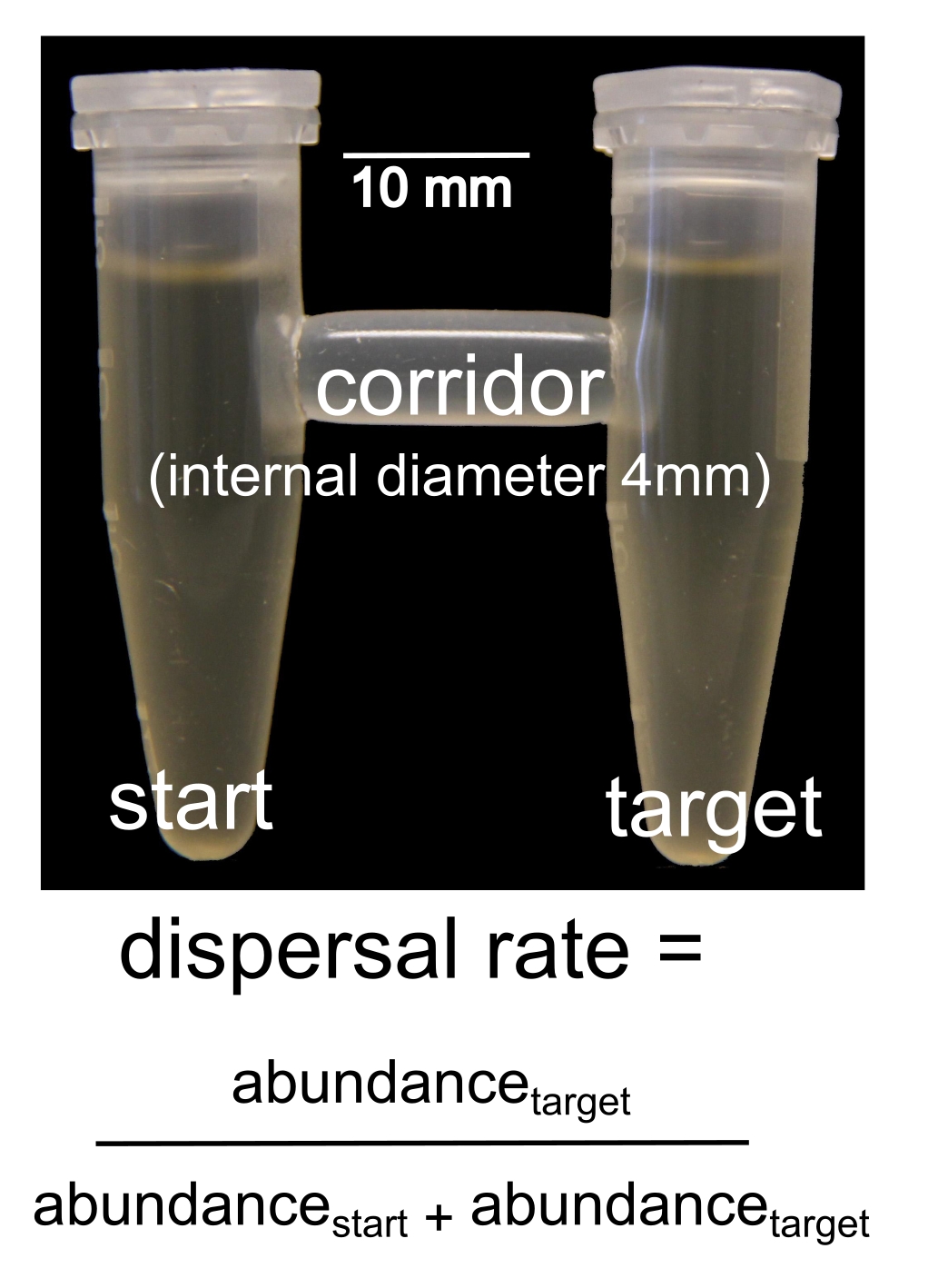

Supplement: Supplemental Information 2 — The formula shows the calculation of the dispersal rate. [file peerj-07-8197-s002.jpg]

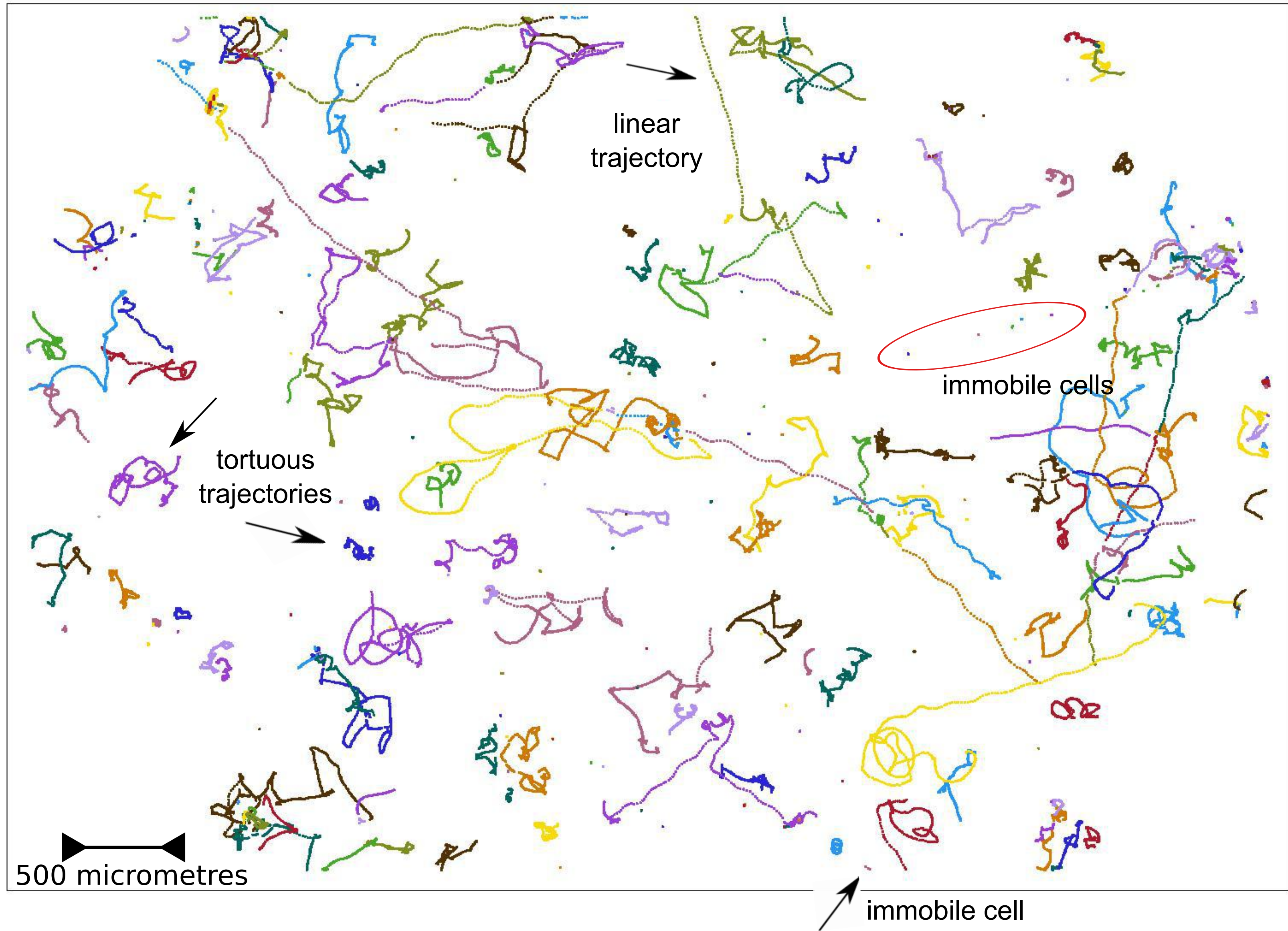

Supplement: Supplemental Information 3 — Different colours show different individual trajectories. The linearity differed among trajectories with some being very linear and others more tortuous (see arrows). Some very short (in time or space) trajectories correspond to non-moving cells. [file peerj-07-8197-s003.pdf]

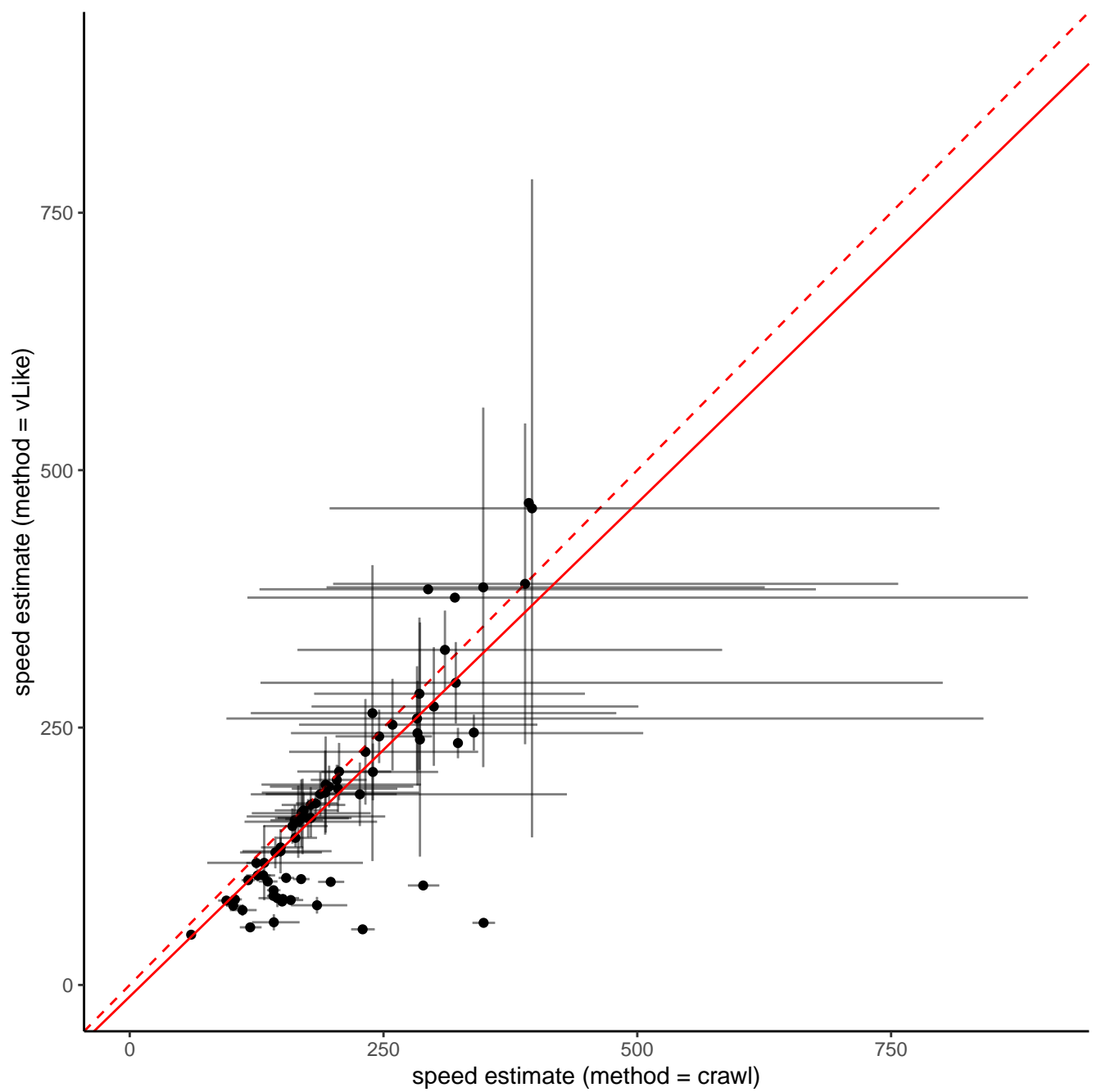

Supplement: Supplemental Information 4 — The dashed red line shows the 1:1 line, while the solid red line is the fit of a reduced major axis regression (accounting for potential error in speed estimates from both exact and approximate methods) fitted to the pairs of speed estimates. The bars show the 95% confidence intervals, whereas the dots are the point estimates. [file peerj-07-8197-s004.pdf]

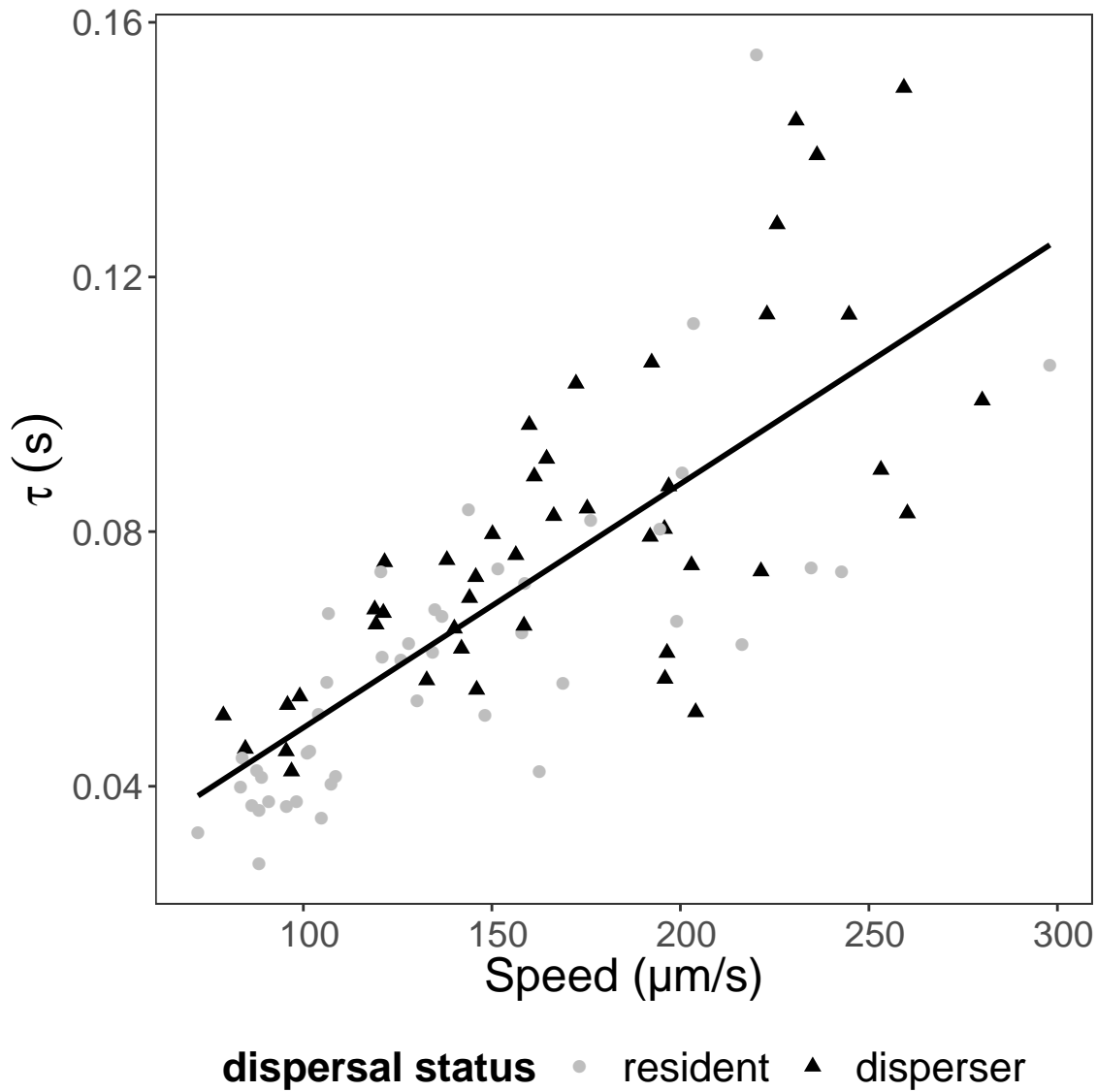

Supplement: Supplemental Information 5 — Faster genotype moved more linear. The strength of the relationship did not differ with dispersal status. [file peerj-07-8197-s005.pdf]

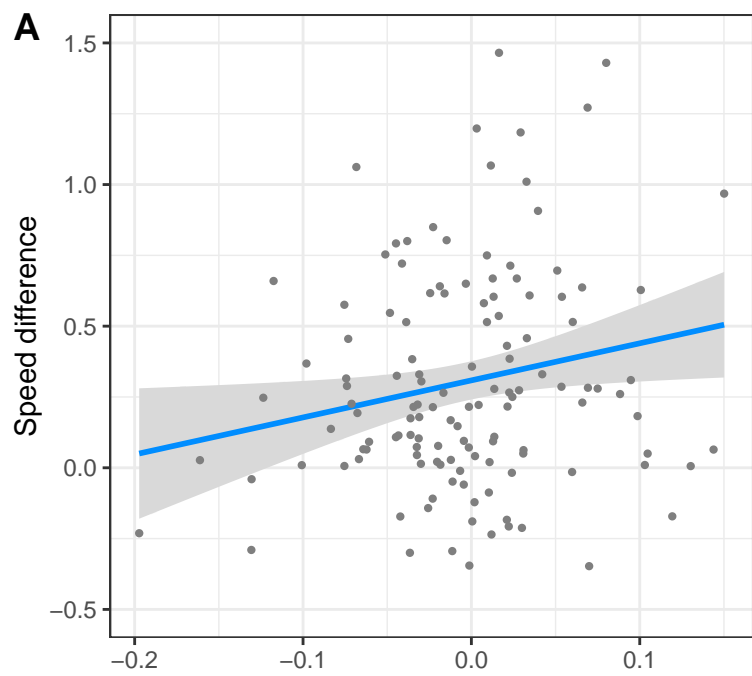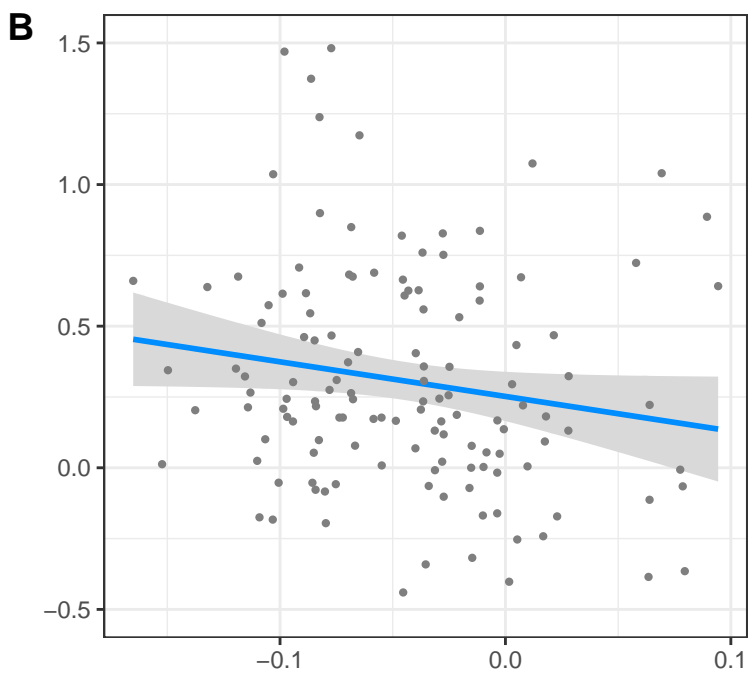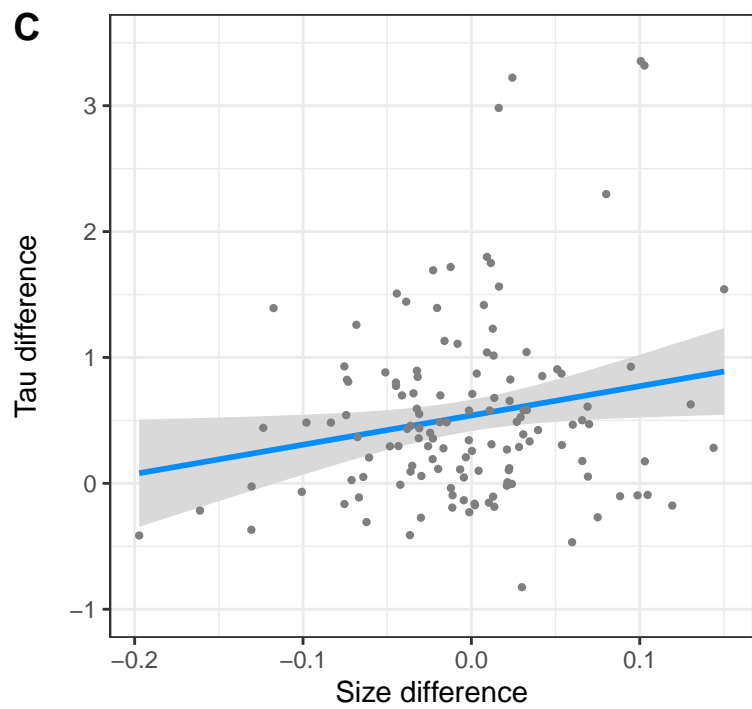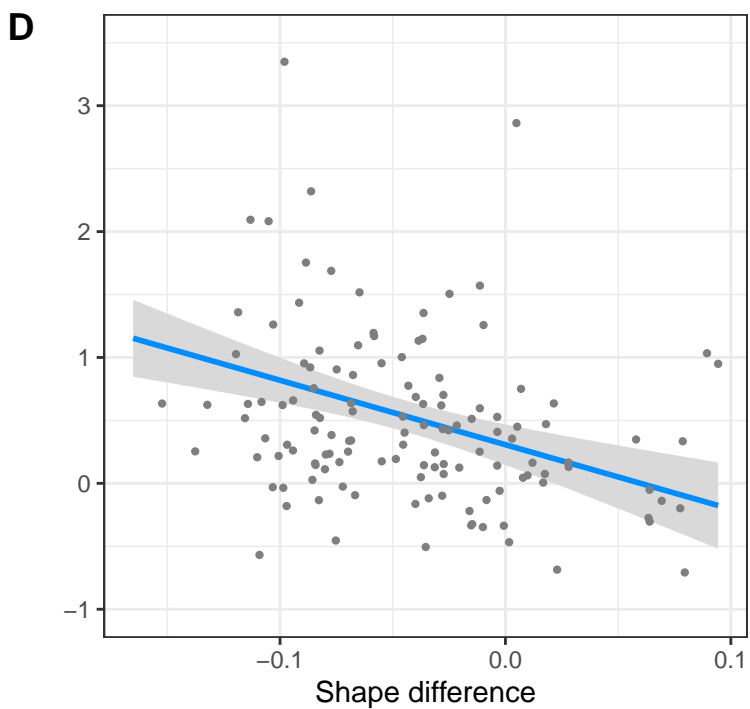

Supplement: Supplemental Information 6 — Genotypes with relatively larger dispersers showed relatively faster and more linear movements. Relatively more elongated dispersers showed relatively slower and less linear movements. [file peerj-07-8197-s006.pdf]
