# Supplementary material for: The interplay between movement, morphology and dispersal in Tetrahymena ciliates
Source: PeerJ. 2019 Dec 17;7:e8197. doi: 10.7717/peerj.8197 (PMC6924321; doi:10.7717/peerj.8197)
Supplement: Supplemental Information 7 — Distributor, isolator and geographic location from which the strain was obtained are listed. [file peerj-07-8197-s007.docx]

| **Our name** | **Distributor and reference** | **Isolator and isolation date** | **Geographic isolation** |
| --- | --- | --- | --- |
| 7 | ATCC 30306 | D.L. Nanney, 1953 | Woods Hole, MA |
| 20 | TSC SD01236 | E. Orias | Laboratory created |
| 4A | CCAP 1630/4A | Nanney & McCoy, 1976 | Unknown |
| B | ATCC 30384 (B-18687) | Simon & Nanney, 1968 | Laboratory created |
| C | TSC SD01216 | Yuhua Shang, 2002 | Laboratory created |
| E | ATCC 205043 | Simon & Nanney, 1986 | McCurdy Pond, ME |
| F | TSC SD00086 | P. J. Bruns | Laboratory created |
| G | TSC SD00112 | Unknown | Unknown |
| H | TSC SD00270 | Unknown | Unknown |
| I | TSC SD01206 | Unknown | Unknown |
| J | TSC SD00626 | Unknown, 1976 | Laboratory created |
| K | TSC SD01590 | Unknown | Laboratory created |
| L | TSC SD01223 | Unknown, 1979 | Laboratory created |
| M | CCAP 1630/1M | Phelps, 1948/9 | Unknown, AZ |
| N | CCAP 1630/1N | Phelps, 1948/9 | Unknown, AZ |
| O | TSC SD01422 (NP1) | L. Rasmussen, 1968 | Laboratory created |
| P | CCAP 1630/1P | Phelps, 1948/9 | Unknown, AZ |
| Q | CCAP 1630/1Q | Phelps, 1948/9 | Unknown, AZ |
| R | TSC SD00703 (SB210) | E. Orias | Laboratory created |
| S | TSC SD01532 | Unknown | Laboratory created |
| T | TSC SD01538 | Unknown | Laboratory created |
| U | CCAP 1630/1U (WH14) | Elliott, 1952 | Woods hole, MA |
| D1 | TSC SD01546 | Doerder, 8/2002 | CRWP, PA |
| D2 | TSC SD01547 | Doerder, 8/2002 | CRWP, PA |
| D3 | TSC SD01548 | Doerder, 06/2003 | SG29, PA |
| D4 | TSC SD01549 | Doerder, 06/2003 | SG29, PA |
| D5 | TSC SD01550 | Doerder, 06/2003 | SG29, PA |
| D6 | TSC SD01551 | Doerder, 06/2003 | SG29, PA |
| D7 | Doerder AK III | Doerder | Unknown |
| D8 | TSC SD01553 | Doerder, 07/2008 | FS136NW, PA |
| D9 | TSC SD01552 | Doerder, 07/2008 | Beaver Meadows, PA |
| D10 | TSC SD01557 | Doerder, 07/2009 | Lake Warren, NH |
| D11 | TSC SD01558 | Doerder, 07/2009 | IslandPond#1, NH |
| D12 | TSC SD01556 | Doerder, 08/2008 | SG69-1, PA |
| D13 | TSC SD01555 | Doerder, 08/2008 | SG69-4, PA |
| D14 | TSC SD01554 | Doerder, 08/2008 | SG69-6, PA |
| D15 | TSC SD01560 | Doerder, 07/2009 | Gregg Lake, NH |
| D16 | TSC SD01559 | Doerder, 07/2009 | Gregg Lake, NH |
| D17 | TSC SD01561 | Doerder, 07/2009 | Willard Pond, NH |
| D18 | TSC SD01562 | Doerder, 07/2009 | Willard Pond, NH |
| D19 | TSC SD01564 | Doerder, 07/2009 | Childs Bog, NH |
| D20 | TSC SD01563 | Doerder, 07/2009 | Russell Reservoir, NH |
| D21 | TSC SD01565 | Doerder, 07/2009 | Perkins Pond, NH |
| D22 | TSC SD01566 | Doerder, 07/2009 | South Pond, VT |

**Table S1:** List of *Tetrahymena thermophila* strains used in this study, as well as the distributor, isolator and geographic location from which the strain was obtained.
