# Supplementary material for: The interplay between movement, morphology and dispersal in Tetrahymena ciliates
Source: PeerJ. 2019 Dec 17;7:e8197. doi: 10.7717/peerj.8197 (PMC6924321; doi:10.7717/peerj.8197)
Supplement: Supplemental Information 8 — The most parsimonious model is shown in bold. K = number of parameters, AICc = Akaike information criterion value, delta = difference with the lowest AIC value, weight = AIC weight. [file peerj-07-8197-s008.docx]

| Model | K | AICc | delta | weight |
| --- | --- | --- | --- | --- |
| **speed ~ disp_status + shape + size + disp_status:shape + 1** | **6** | **2846.68** | **0** | **0.69** |
| speed ~ disp_status + shape + size + disp_status:shape + disp_status:size + 1 | 7 | 2848.72 | 2.04 | 0.25 |
| speed ~ disp_status + shape + disp_status:shape + 1 | 5 | 2851.37 | 4.68 | 0.07 |
| speed ~ disp_status + shape + size + 1 | 5 | 2861.1 | 14.41 | 0 |
| speed ~ disp_status + shape + size + disp_status:size + 1 | 6 | 2863.19 | 16.51 | 0 |
| speed ~ disp_status + size + 1 | 4 | 2863.27 | 16.58 | 0 |
| speed ~ disp_status + shape + 1 | 4 | 2865.25 | 18.56 | 0 |
| speed ~ disp_status + size + disp_status:size + 1 | 5 | 2865.35 | 18.66 | 0 |
| speed ~ disp_status + 1 | 3 | 2866.73 | 20.05 | 0 |
| speed ~ size + 1 | 3 | 2881.54 | 34.86 | 0 |
| speed ~ shape + size + 1 | 4 | 2883.29 | 36.6 | 0 |
| speed ~ 1 | 2 | 2884.02 | 37.33 | 0 |
| speed ~ shape + 1 | 3 | 2885.86 | 39.18 | 0 |
