# Supplementary material for: The interplay between movement, morphology and dispersal in Tetrahymena ciliates
Source: PeerJ. 2019 Dec 17;7:e8197. doi: 10.7717/peerj.8197 (PMC6924321; doi:10.7717/peerj.8197)
Supplement: Supplemental Information 9 — The most parsimonious model is shown in bold. K = number of parameters, AICc = Akaike information criterion value, delta = difference with the lowest AIC value, weight = AIC weight. [file peerj-07-8197-s009.docx]

| Model | K | AICc | delta | weight |
| --- | --- | --- | --- | --- |
| **tau ~ disp_status + shape + disp_status:shape + 1** | **5** | **-1129.43** | **0** | **0.58** |
| tau ~ disp_status + shape + size + disp_status:shape + 1 | 6 | -1128.16 | 1.27 | 0.31 |
| tau ~ disp_status + shape + size + disp_status:shape + disp_status:size + 1 | 7 | -1126.06 | 3.37 | 0.11 |
| tau ~ disp_status + shape + 1 | 4 | -1114.82 | 14.6 | 0 |
| tau ~ disp_status + shape + size + 1 | 5 | -1113.47 | 15.96 | 0 |
| tau ~ disp_status + shape + size + disp_status:size + 1 | 6 | -1111.54 | 17.89 | 0 |
| tau ~ disp_status + 1 | 3 | -1106.99 | 22.44 | 0 |
| tau ~ disp_status + size + 1 | 4 | -1105.33 | 24.1 | 0 |
| tau ~ disp_status + size + disp_status:size + 1 | 5 | -1103.44 | 25.99 | 0 |
| tau ~ 1 | 2 | -1079.59 | 49.84 | 0 |
| tau ~ shape + 1 | 3 | -1079.1 | 50.32 | 0 |
| tau ~ size + 1 | 3 | -1077.73 | 51.7 | 0 |
| tau ~ shape + size + 1 | 4 | -1077.29 | 52.14 | 0 |
