# Supplementary material for: The interplay between movement, morphology and dispersal in Tetrahymena ciliates
Source: PeerJ. 2019 Dec 17;7:e8197. doi: 10.7717/peerj.8197 (PMC6924321; doi:10.7717/peerj.8197)
Supplement: Supplemental Information 10 — The most parsimonious model is shown in bold. K = number of parameters, AICc = Akaike information criterion value, delta = difference with the lowest AIC value, weight = AIC weight. [file peerj-07-8197-s010.docx]

| Model | K | AICc | delta | weight |
| --- | --- | --- | --- | --- |
| speed_diff ~ shape_diff + size_diff + shape_diff:size_diff + 1 | 5 | 129.54 | 0 | 0.46 |
| **speed_diff ~ shape_diff + size_diff + 1** | **4** | **130.32** | **0.78** | **0.31** |
| speed_diff ~ size_diff + 1 | 3 | 131.99 | 2.45 | 0.13 |
| speed_diff ~ shape_diff + 1 | 3 | 133.43 | 3.89 | 0.07 |
| speed_diff ~ 1 | 2 | 134.87 | 5.33 | 0.03 |
