# Supplementary material for: The interplay between movement, morphology and dispersal in Tetrahymena ciliates
Source: PeerJ. 2019 Dec 17;7:e8197. doi: 10.7717/peerj.8197 (PMC6924321; doi:10.7717/peerj.8197)
Supplement: Supplemental Information 11 — The most parsimonious model is shown in bold. K = number of parameters, AICc = Akaike information criterion value, delta = difference with the lowest AIC value, weight = AIC weight. [file peerj-07-8197-s011.docx]

| Model | K | AICc | delta | weight |
| --- | --- | --- | --- | --- |
| tau_diff ~ shape_diff + size_diff + shape_diff:size_diff + 1 | 5 | 290.04 | 0 | 0.62 |
| **tau_diff ~ shape_diff + size_diff + 1** | **4** | **291.51** | **1.47** | **0.3** |
| tau_diff ~ shape_diff + 1 | 3 | 294.22 | 4.18 | 0.08 |
| tau_diff ~ size_diff + 1 | 3 | 307.8 | 17.76 | 0 |
| tau_diff ~ 1 | 2 | 309.7 | 19.65 | 0 |
